# Anesthetic management for emergency cesarean section in a patient with status epilepticus: A case report

**DOI:** 10.1097/MD.0000000000036331

**Published:** 2023-12-01

**Authors:** Liyuan An, Min Gao, Guoning Su, Hua Li, Liping Tao, Danxia Lu, Yan Qu

**Affiliations:** a Department of Anesthesiology, Affiliated Hospital of Yunnan University, Kunming, PR China.

**Keywords:** epilepsy, preeclampsia, anesthetic management, case report

## Abstract

**Rationale:**

The presence of clinically significant repeated maternal epilepsies during pregnancy increases the risk of adverse maternal, fetal and neonatal outcomes. However, there are few guidelines for anesthesiologists to deal with this situation.

**Patient concerns and Diagnoses:**

A 28-year-old primigravida was transferred to the operating room for emergency cesarean section. Based on the patient’s complaints and clinical appearance, provisional diagnosis of preeclampsia at 33 weeks’ gestation as well as frequent and repeated grand mal convulsions at 14 years of age were considered. The anesthetic modalities of the disease are also discussed.

**Interventions and outcomes:**

Because the usual antiepileptic therapy had failed, the patient with status epilepticus underwent surgery under general anesthesia. The newborn was handed to the pediatrician and the patient was transferred to the intensive care unit for further observation and discharged 4 days later. No peri-operative or anesthetic complications were observed.

**Lessons:**

Providing anesthesia to patients undergoing cesarean section poses major challenges for anesthesiologists. Close monitoring and proper treatment can help reduce risks for both the mother and baby.

## 1. Introduction

Seizure disorders are the most frequent neurological complication associated with pregnancy. Approximately 1% of pregnant women experience seizure.^[1]^ The presence of clinically significant repeated maternal epilepsies during pregnancy increases the risk of adverse maternal, fetal and neonatal outcomes. In this case, a 28-year-old parturient with repeated grand mal convulsions and severe preeclampsia underwent emergent cesarean section with an excellent outcome. The anesthetic management of this patient is also discussed.

While the obstetrical literature has a rather complete exposition of epilepsy associated with pregnancy, scant attention of the anesthesiologist is given to status epilepticus for patient and infant safety. Perinatal emergencies create physiological challenges and trigger intrinsic survival. The resultant fetal hypoxemia may also stress the fetus in initiating labor. During extensive oxygen desaturation and decompensation, the focus should be on maternal stabilization, which will subsequently enhance fetal stabilization. Clinical assessments, critical thinking, and decision-making must be quick and appropriate to increase the likelihood of a positive outcome for the mother, fetus, and neonate. Here, we discuss the case of a woman with epilepsy during the periconceptional period.

## 2. Case presentation

A 28-year-old primigravida at 33 weeks of gestation was hospitalized with progressive blood pressure suggestive of preeclampsia as well as frequent and repeated grand mal convulsions. She was first diagnosed with hypertension at 6 months of gestation but refused any treatment. A few days later, the patient was referred to the emergency department after experiencing unprovoked convulsions. She was given labetalol 50 mg twice a day. The anti-epilepsy drugs were adjusted to levetiracetam 2 tablets twice a day, lamotrigine tablets 25 mg once a day, and quetiapine fumarate tablets 0.05 g once a day.

The patient’s history revealed that she had an epileptic seizure at the age of 14 years, while carbamazepine and oxcarbazepine therapies had been instituted. However, the patient had taken these drugs irregularly. The patient was diagnosed with hydrocephalus at birth. Neurological examination revealed findings suggestive of organic mental disorders of the brain. A computed tomography scan of her brain before pregnancy showed mild dilatation of the bilateral lateral ventricles. General physical and routine laboratory examinations were not regularly performed.

The prenatal course was uneventful when she experienced several fainting spells. Shortly after admission to the hospital she developed repeated convulsions and remained comatose between seizures. The temperature was 36.7 °C, pulse was rapid (140) and of good volume, and blood pressure was 138/109 mm Hg. The heart and lungs were normal. There was no evidence of edema. The abdomen was enlarged to the size of gestation, and the fetal heart tones were normal. Obstetric ultrasound revealed that the fetus was normally developed and in a transverse lie. Preoperational assessment revealed no preexisting cardiorespiratory dysfunction. Other laboratory results were normal. Neurological examination failed to reveal any localizing signs in the central nervous system. The deep tendon reflexes were exaggerated. No signs of papilledema or meningitis were observed.

She experienced 2 convulsions 4 days after hospitalization. She was administered 10 mg intravenous diazepam immediately, followed by 100 mg labetalol every 8 hours and 30 mg nifedipine tablets to control her blood pressure between 120–148/80–109 mm Hg. Antiepileptic medications were administered. Lamotrigine was added to the previous therapy regimen. In addition, oxygen and glucose and water infusions were administered.

For the next 6 hours, the seizures increased in frequency and severity; despite sedation, she experienced convulsions almost every 2 hours necessitating immediate cesarean section. Because the usual antiepileptic therapy had failed, the patient was transferred to the operating room for emergency cesarean section after treatment with 5 mg midazolam. At this time, the patient became restless and unconscious with closed teeth. After discussion with the obstetrician, we planned to administer general anesthesia. Routine noninvasive monitoring includes noninvasive blood pressure, heart rate, pulse oximetry, and electrocardiography (ECG). Her baseline blood pressure and heart rate were 130/95 mm Hg and 125/min, respectively. Her pulse oximetry was 92% while breathing room air, and the ECG results showed a normal sinus rhythm. After propofol 150 mg, remifentanil 50 µg and rocuronium 40 mg were administered intravenously, endotracheal intubation was performed, and surgery was started.

A female baby was delivered 5 minutes after tracheal intubation, and there were no maternal complications. The Apgar scores for the newborn infants were 5 at 1 minute and 2 at 5 minutes. After neonatal endotracheal intubation for oxygenation, the Apgar score was 6 at 10 minutes. The newborn was then transferred to a pediatrician. Propofol and remifentanil were administered to maintain general anesthesia. The surgery lasted 50 minutes and the patient’s BP remained 110–120/60–70 mm Hg during the operation. The intra-operative blood loss was estimated to be 400 mL. Hartmann’s solution (1100 mL) and 6% Hydroxyethyl Starch 130/0.4 (500 mL) were used to maintain maternal blood pressure. Following delivery, oxytocin 20 IU was administered via a slow intravenous injection. The patient was transferred to the intensive care unit for further observation and sent back to the obstetric unit 1 day later in conscious and stable condition. No peri-operative or anesthetic complications were observed. The patient was discharged 4 days later.

## 3. Discussion

Intrapartum emergencies, such as seizures, amniotic fluid embolus, hemorrhage, and uterine rupture, are challenging for all anesthesiologists and obstetricians because of the increased risk of adverse outcomes for the mother and fetus. Epilepsy, characterized by recurrent seizures, has an annual prevalence of 40 to 80 per 100,000 individuals worldwide.^[2,3]^ It is estimated that 0.3% to 0.7% of pregnancies are carried by women with epilepsy in developed countries.^[4,5]^ After excluding other causes, eclampsia was found to be the most frequent cause of seizures in obstetric patients. Other potential causes of seizures during pregnancy include amniotic fluid embolism, water intoxication, and local anesthetic toxicity. Acute hyponatremia (especially < 125 mmol/L) can cause confusion and seizures, resulting from brain cell edema.^[6]^ Hyponatremia can also lead to neonatal seizures.^[7]^ During the antepartum, intrapartum, or postpartum period, seizures may occur. Headache/migraine, scotoma, visual abnormalities, and hyperreflexia are common symptoms of the central nervous system that may precede an episode of eclampsia.

Responses to seizure activity in the mother and fetus may range from mild to severe. Although it is a common observation, apnea is often tolerated by pregnant women, with minimal morbidity. Pathological uterine reactions include placental abruption, severe vasospasm, and uteroplacental insufficiency. This may stimulate a survival response from the fetus, as evidenced by tachycardia, bradycardia, deceleration, or decreased variability. The risk of maternal-fetal compromise is related to the duration of seizures. The maternal-fetal response may be delayed or continued long after the seizure. However, given the extensive and expanding use of antiepileptic drugs (AEDs) for pain and mental problems, the percentage of pregnant women taking AEDs may be substantially higher.^[8]^ AEDs are mostly central depressants that have synergistic effects with narcotic analgesics and sedatives.

Furthermore, it is unclear what triggers eclampsia precisely. Theories include arterial vasoconstriction, cerebral edema, ischemia, hemorrhage, hypertensive encephalopathy, or vasospasms.^[9,10]^ Normal vasoconstriction fails and blood flow increases in patients with severe hypertension. Vessel dilation leads to ischemia, increased permeability, plasma shift, and cerebral edema. These changes may be confirmed in as many as 90% of patients.^[11]^ Plasma shifts and cerebral edema are caused by dilated, ischemic, and permeable vessels. As many as 90% of eclamptic individuals may have these alterations verified. The management of eclampsia involves maternal stabilization followed by prompt birth. Magnesium sulfate is the preferred drug for eclampsia and seizure prophylaxis.^[12]^ In addition, antihypertensive medication (hydralazine or labetalol) may be administered to stabilize maternal diastolic pressure if it is above 105–110 mm Hg. Women receiving intravenous magnesium sulfate infusions are at risk of magnesium toxicity. Loss of deep tendon reflexes is an early sign of such toxicity. Frequent blood pressure assessments are essential for evaluating trends. Anesthetists must be proactive in expecting maternal complications of preeclampsia, planning for possible emergent cesarean birth and allocating adequate equipment as well as extra staff for neonatal stabilization.

Therefore, an individualized approach is required. All discussions with pregnant women should include a survey of preeclamptic central nervous system indications. Extraordinary hypertension puts pregnant women at the highest risk of seizures or coma. Pregnant women with a history of seizures require close monitoring for recurrences. The primary goal is to ensure an adequate oxygen supply to the mother and fetus. This case describes the management of a pregnant patient with severe seizures, compounded by the presence of preeclampsia. Our anesthetic objectives were to secure the patient’s airway and maintain stable circulation. Hypertension would have produced preeclampsia and eclampsia, not only increasing maternal oxygen consumption but also decreasing fetal oxygenation, which is a critical factor in epileptic seizures.

Administering anesthesia for cesarean section in patients with grand mal seizures presents a major challenge to anesthesiologists. This is due to the rarity of emergency cases in pregnancy and labor, as well as the lack of standard protocols for anesthetic management. When first seeing a patient with these conditions, it is important for the anesthetist to remain calm. Combined spinal-epidural anesthesia is commonly used for cesarean section, but it may be challenging to perform in patients with certain physical/mental conditions, such as epilepsy. Furthermore, intrathecal anesthesia poses a high risk for pregnant preeclamptic patients because their airway cannot be properly assessed before emergency surgery.

There are several concerns regarding the anesthetic technique, including the potential for precipitation of seizure activity. Upon reviewing the literature on anesthetic dosages for epileptic patients, we found that our usual dosages may not be suitable or safe. the Based on our experience, we believe that rapid delivery of the fetus is important for managing hemodynamic deterioration and correcting preeclampsia in the mother. The first step was to assess the condition of the parturients. After the parturient enters the operating room, she immediately receives ECG and pulse oximetry monitoring and prepares antiepileptic and emergency drugs. Ensure at least 2 venous accesses for rapid infusion of AEDs. Choose either epidural block or general anesthesia for induction based on assessment to prevent epileptic seizures.

**Figure 1.**
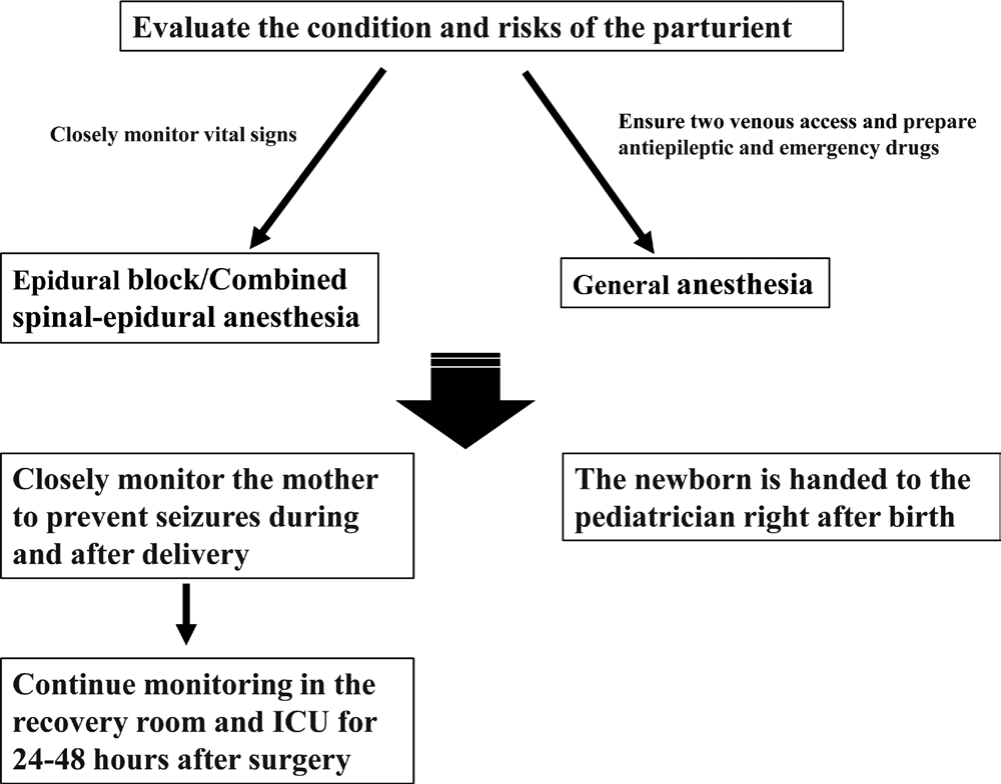
Epileptic parturient anesthesia flowchart.

Some studies conducted by Chinese experts suggested that the type of epilepsy did not significantly influence the likelihood of seizures during pregnancy or the outcomes. However, pregnant women with epilepsy who also had hypertensive disorders were more prone to seizures than those without hypertensive disorders.^[13]^ Pregnant women with epilepsy generally face higher risks of various complications such as anemia, gestational hypertension, premature rupture of membranes, cesarean delivery, postpartum hemorrhage, lower birth weight, and Apgar score. The choice of anesthetic agents and doses must consider the impact on neonates already compromised by maternal seizures and preeclampsia. Seizures also significantly increase the mother’s metabolic demands, while preeclampsia reduces the oxygen supply, necessitating aggressive fluid resuscitation and blood pressure control to maintain perfusion of vital organs in both patients. Managing these complex cases requires a collaborative approach across specialties, with coordination among anesthesiology, obstetrics, neurology and pediatrics teams being key to optimizing outcomes. Following delivery, the risks of recurrent seizures, aspiration pneumonia, and worsening preeclampsia warrant admission to intensive care unit for close monitoring and management (Fig. 1).

In summary, this case illustrates the substantial challenges and important considerations in providing anesthesia and perioperative care to pregnant women with epilepsy and preeclampsia. A multidisciplinary team approach is essential to improve outcomes for both the mother and neonate in high-risk and highly complex situations. The lessons learned from this case report can help inform the future management of similar complex obstetric cases. Close monitoring and proper treatment can help reduce risks for both the mother and baby.

## Author contributions

**Data curation:** Danxia Lu.

**Resources:** Hua Li, Liping Tao.

**Supervision:** Yan Qu.

**Visualization:** Min Gao.

**Writing – original draft:** Liyuan An.

**Writing – review & editing:** Guoning Su.
